# Detecting Wildfire Smoke Onset from Fixed View Camera Networks

**DOI:** 10.3390/s26196020

**Published:** 2026-09-23

**Authors:** Shakhnoza Muksimova, Sabina Umirzakova

**Affiliations:** 1 Department of Computer Engineering, Gachon University, Seongnam-si 13120, Gyeonggi-do, Republic of Korea; 2 Department of Computer Science, Chungbuk National University, Cheongju-si 28644, Chungcheongbuk-do, Republic of Korea

**Keywords:** smoke onset detection, earliness aware learning, time to detection, scene dynamics prior, video anomaly detection, emergence field, zero-shot cross-dataset evaluation, fixed-view camera networks

## Abstract

(1) Background: A wildfire smoke detector’s practical worth is counted in minutes, not percentage points: an alarm raised ten minutes after ignition is worth far less than one raised at two, whatever the two systems’ frame-level scores suggest. Time to detection, however, is almost always computed after training rather than optimized during it. (2) Methods: We treat incipient smoke not as an object to be recognized but as an onset to be caught: a faint, spatially coherent structure that thickens monotonically. At the same time, the rest of the scene continues behaving as it always has. The proposed study learns that habitual behavior from unlabeled fire-free video, surfaces departures from it as a per-tile prediction residual, and reads those residuals through an emergence field of four inspectable cues: residual magnitude, opacity accretion, motion coherence, and temporal persistence. Each common distractor fails a different cue, and detection delay and false alarms directly contribute to the loss. (3) Results: On FIgLib, the proposed study raises its first alarm 1.52 min after ignition while holding 0.21 false alarms per camera-day, and carries over to PyroNear-2025 and SKLFS-WildFire without labels from either. (4) Conclusions: Treating earliness as an optimization target rather than a reporting statistic yields detectors that stay both early and quiet on cameras they never saw in training.

## 1. Introduction

The frequency, extent, and severity of wildfires have risen markedly over the past two decades, driven by warmer temperatures, prolonged drought, and increasingly fire-prone weather [1,2]. Formal attribution studies now link a growing share of global burned area, and of the most energetically extreme fire events, directly to anthropogenic climate change [1,2], and extreme regional fire years have become substantially more probable under the contemporary climate [3,4]. These trends are not confined to a single region. Parts of Europe face up to a tenfold increase in the likelihood of extreme fire weather [5], and recent seasons have repeatedly produced record-breaking losses across multiple continents. On high-risk days, a small ignition can escalate beyond control within minutes, so the interval between ignition and the first credible alarm, the time to detection, directly bounds the effectiveness of any response. Fixed view camera networks now provide continuous imagery over large wildland areas, making automated visual detection an attractive complement to sensor- and satellite-based systems [6,7]. The central difficulty is that the frames that matter most, those captured moments after ignition, contain smoke that is thin, low in contrast, and easily confused with clouds, haze, fog, dust, and illumination artifacts. Comprehensive surveys of the field consistently identify false alarms, early-stage sensitivity, and cross-domain generalization as central and still open problems [6,7].

Most deep learning systems approach early detection as an appearance-recognition problem. A detector and classifier are applied to a single frame, a short pair of frames [8], and temporal information, when used at all [9], is folded in through a recurrent and channel concatenation mechanism spanning only a couple of time steps. Time to detection is then reported as an evaluation statistic rather than being part of the objective the model actually minimizes [10,11]. Three limitations follow: first, the temporal context is too short to capture the slow [12], minutes-long emergence that most distinguishes early smoke from static appearance [13]. Recent work that lengthens the temporal horizon reports meaningful gains in early-warning latency [14]. Second, the temporal aggregator is opaque and carries no inductive bias toward the physical signature of smoke, so it readily latches onto distractor motion. Third, because the loss optimizes frame accuracy rather than earliness, models are not explicitly pushed to alarm as soon as evidence is sufficient. We propose Earliness-Aware Residual Learning (EARL), which reframes early smoke detection as the detection of an emergence onset and optimizes directly for detecting it early. EARL learns normal scene dynamics from abundant fire-free footage and identifies smoke onset through deviations from this learned behavior. Our contributions are:

- A self-supervised scene dynamics prior that learns per-camera background evolution from abundant unlabeled fire-free footage, converting the chronic scarcity of early-stage labels into an advantage and exposing smoke onset as a structured prediction residual. This connects the label-scarce, latency-first wildfire setting to the broader literature on learning normal dynamics for anomaly detection.
- An interpretable emergence field that replaces black-box temporal aggregation with four physically motivated signals: residual magnitude, monotonic opacity growth, motion coherence, and temporal persistence, each of which real early smoke satisfies, and common false-positive triggers violate. Unlike prior temporal detectors, these signals are explicit and inspectable, in the spirit of interpretable prediction error and appearance motion consistency descriptors.
- An earliness-aware objective adapted from early time series classification that in concert rewards correct detection, penalizes latency after ignition, and suppresses hard negative false alarms. The training objective explicitly incorporates time to detection as an optimization target. This directly targets the false alarm and early-stage sensitivity gaps that surveys and dataset builders repeatedly flag as decisive for operational value.
- A latency-first evaluation protocol reporting time to detection, an earliness recall curve, false alarms per camera-day, and cross-dataset generalization across publicly available datasets, together with an ablation design that isolates the contribution of each component.

## 2. Related Works

Research on visual wildfire detection has evolved considerably, moving from handcrafted color and motion cues toward deep learning approaches spanning classification, object detection, segmentation, and temporal analysis. Alongside this methodological shift, recent work has contributed larger wildfire datasets and evaluation settings that place greater weight on early-stage smoke, difficult negative samples, and cross-domain generalization, reflecting a growing recognition that laboratory accuracy alone does not guarantee operational value [15]. In parallel, advances in temporal modeling and self-supervised video anomaly detection offer principled mechanisms for exploiting change across consecutive observations and for flagging deviations from a scene’s normal dynamics, both of which are central to detecting smoke as it first emerges [16]. Building on these developments, this section reviews four closely related areas that inform our approach, namely vision-based fire and smoke detection, wildfire smoke benchmarks, temporal detection models, and self-supervised prediction and anomaly modeling [17].

### 2.1. Vision-Based Fire and Smoke Detection

Early systems relied on hand-crafted color and motion cues, which are efficient but brittle under varied illumination and background clutter. Convolutional networks subsequently improved robustness for both classification and segmentation, and single-stage detectors of the YOLO family became the mainstream choice for localizing fire and smoke in real time. Recent variants continue to push multi-scale accuracy and efficiency through improved feature aggregation and lightweight backbones [8,11,12]. More recent work applies vision transformers and CNN-transformer hybrids to capture global context alongside fine local detail. Swin transformer-based smoke detectors improve robustness to cloud and haze interference [10], and multi-scale remote sensing networks combine synthetic pretraining with adaptive spatial aggregation for forest-fire targets [9]. Promotable and transformer-based segmentation models have also been explored for pixel-level plume delineation, including UAV-oriented segmentation that additionally interprets the fire front [18] and dedicated smoke segmentation datasets that support such models [19]. A recurring practical obstacle is the scarcity and limited diversity of early-stage annotations, which has motivated multifaceted synthetic datasets that expand coverage of terrain, weather, and illumination [20]. Comprehensive surveys and reviews document this progression from handcrafted features to data-driven frameworks and consistently identify false alarms, early-stage sensitivity, and generalization as the field’s open problems [6,7]. Systems designed explicitly around hard negative mining and diverse negative sampling report reduced false alarms on smoke-like confusers such as fog, clouds, and reflections, underscoring that false-alarm control is as central as raw accuracy [21].

### 2.2. Benchmarks for Wildfire Smoke

The Fire Ignition Library (FIgLib) provides sequences from fixed Southern California cameras of the HPWREN network, with frames both before and after ignition, enabling evaluation of ignition time detection and TTD at a one-minute cadence [13]. PyroNear-2025 contributes a large, in-the-wild collection of images and videos spanning multiple countries and, unlike most predecessors, includes video suitable for sequential modeling. Its authors report a deliberately challenging cross-dataset F1 near 60% [22]. SKLFS-WildFire has been released as a large real-world test set drawn from thousands of ground-mounted-camera videos, with a strict geographically disjoint train/test split and a large pool of difficult negatives, making it well suited to systematic evaluation and to measuring false alarms at operational volume [21]. Complementary synthetic and multi-scenario datasets such as M4SFWD further broaden the range of terrains, weather conditions, and lighting levels available for training [20]. Together, these define a train, validation, cross-test regime that our protocol adopts, partitioning by fire event and by camera so that no scene leaks between training and evaluation.

SmokeyNet, the most influential temporal architecture on FIgLib, combines a convolutional backbone, a recurrent aggregator, and a transformer over tiled frames, using two frames of context, and reports TTD on the order of a few minutes, with intermediate supervision stabilizing training [13]. Related efforts pair detectors with recurrent classifiers and move toward longer, transformer-based temporal reasoning. Recursive transformer models that fuse multi-type and multi-source information have pushed early detection latency toward the ten-minute horizon [14], and multimodal early warning models couple smoke cues with auxiliary signals for risk assessment [15]. A recurring pattern in this line of work is short temporal context, often a two-frame concatenation, paired with objectives that target frame accuracy. Authors of subsequent systems have explicitly noted that a more refined temporal model was left to future work. EARL addresses this gap by lengthening and physically structuring the temporal representation and by making earliness an explicit objective.

### 2.3. Self-Supervised Prediction and Anomaly Modeling

EARL’s scene dynamics prior is methodologically closest to the video anomaly detection paradigm, in which a model learns regular patterns from footage containing only normal behavior and treats deviations from those patterns as anomalies [16]. Two design choices in that literature are directly relevant to early smoke. First, forecasting future appearance and feature evolution and scoring the prediction residual provides a label-free onset signal. Memory-guided event prediction [23], multi-frame prediction-error framing [24], bidirectional skip frame prediction [25], and consistency-based formulations [17] all rely on the fact that abnormal onsets. Second, appearance-motion synergy and error metrics tuned to structural change rather than raw intensity improve sensitivity to subtle, low-contrast deviations [23,26], the regime of incipient smoke. EARL adapts these ideas to the fixed camera wildfire setting [27]. It predicts per-tile background feature evolution from abundant fire-free footage [28], calibrates the residual per camera and time of day [29], and reads the resulting anomaly field through the physically motivated signals of the emergence field, so that only unordinary, coherent, monotonically growing structure raises an alarm [30].

## 3. Proposed Method

We propose EARL, a temporal framework for early wildfire smoke detection from fixed-view camera sequences. The method is designed to identify smoke in its earliest stage of development by modeling deviations from a scene’s normal dynamics and tracking how those deviations evolve across space and time. Because the operative evidence at onset is often small and faint, each frame is divided into spatial tiles, which preserves localized cues that a global representation would tend to wash out. EARL comprises four main components. The first is a self-supervised scene dynamic prior trained on fire-free footage, which learns what a given camera normally looks like and thereby exposes smoke onset as a departure from that expectation. The second is an interpretable emergence field that characterizes residual magnitude, opacity growth, motion coherence, and temporal persistence. The third is a factorized temporal spatial attention module that aggregates information across frames and regions Figure 1. The fourth is an earliness-aware objective that folds detection latency and false alarm control directly into training. The following subsections describe each component in detail.

### 3.1. Self-Supervised Scene Dynamics Prior

The frames most decisive for early detection are those in the first minutes after ignition, because ignition events are rare and their earliest smoke is faint and ambiguous even to human annotators. Fire-free footage from the same fixed cameras, by contrast, is effectively unlimited. EARL converts this asymmetry into a learning signal. The scene prior is trained on abundant fire-free footage to model normal camera-specific dynamics, allowing smoke onset to be identified as a deviation from the learned background behavior. This is a predictive, self-supervised pretext task that requires no smoke labels. Let a fixed camera produce frames $I_{1},\;I_{2},\ldots ,$ each partitioned into an N-tile grid so that small, localized plumes are not diluted by full frame pooling. Write $x_{t,i}$ for tile i of frame t. A shared convolutional backbone $f_{\theta }$ maps each tile to a feature vector:

(1) $$z_{t,i}=f_{\theta }\left(x_{t,i}\right)\in R^{d}$$

To supply prediction targets without inviting representation collapse, we maintain a second encoder $f_{\xi }$ whose weights are an exponential moving average (EMA) of $f_{\theta }$ it produces target features ${\tilde{z}}_{t,i}=f_{\xi }\left(x_{t,i}\right)$ that receive a stop gradient. Over a context window of length W, the predictor $g_{\phi }$ forecasts the next frame’s tile features:

(2) $${\tilde{z}}_{t,i}=g_{\phi }\left({\left\{z_{t-W:t-1,j}\right\}}_{j=1}^{N},e_{c},\tau_{t}\right)$$

Here $g_{\phi }$ is a compact temporal model that attends over both the tile’s own recent history and its spatial neighborhood $e_{c}$ is a learned camera identity embedding, and $\tau_{t}$ is a time of day. On fire-free windows only $g_{\phi }$ and $nf_{\theta }$ are trained to minimize a normalized feature regression loss between the forecast and the target:

(3) $$L_{pred}=\frac{1}{N}\sum_{i=1}^{N}\left[1-cos\left({\hat{z}}_{t,i},sg\left({\tilde{z}}_{t,i}\right)\right)\right]$$

Prediction is performed in feature space to emphasize semantic and slowly varying scene structure. Feature space forecasting reduces sensitivity to high-frequency texture and focuses the prediction model on semantic and slowly varying structural changes associated with early smoke. Because the objective is defined entirely on fire-free footage, the recurring dynamic distractors that plague fire detectors, drifting clouds, swaying foliage, forming and dissipating haze, and the diurnal illumination cycle, become part of the predicted normal and therefore produce small residuals. Conditioning on $\tau_{t}$ specifically absorbs dawn glare and low-light regimes that would otherwise masquerade as anomalies. The prior thus models the scene’s ordinary variability, so that only unordinary evolution stands out.

At inference, the per-tile residual is the gap between what actually happened and what the prior expected $r_{t,i}=\;{\tilde{z}}_{t,i}-\;{\hat{z}}_{t,i}$. A raw residual norm is not directly comparable across tiles; a tile framing a busy tree line is intrinsically noisier than one framing open sky. We therefore calibrate per tile, per camera, and per time-bin, using residual statistics estimated over fire-free data. A mean $\mu_{c,i}$ and covariance from which the onset score is the Mahalanobis distance:

(4) $$a_{t,i}=\sqrt{{\left(r_{t,i}-\mu_{c,i}\right)}^{⊺}\sum_{c,i}^{-1}\left(r_{t,i}-\mu_{c,i}\right)}$$

### 3.2. The Emergence Field

The emergence field provides an explicit temporal descriptor composed of four physically motivated signals: residual magnitude, opacity growth, motion coherence, and temporal persistence. Each principal false positive category violates at least one. Throughout, let the window ending at t span frames $k\in \left\{t-W+1,\ldots ,t\right\}$, and let N(i) denote the spatial neighborhood of tile i. Three of the four signals build on the fact that smoke is a semi-transparent, accreting veil. It reduces local contrast and adds a gray haze rather than replacing the background outright. We therefore attach a lightweight opacity head $h_{\psi }$ that maps the tile’s features and its residual to a soft veiling estimate, trained combined with the detection heads:

(5) $$o_{t,i}=h_{\psi }\left(z_{t,i},r_{t,i}\right)\in \left[0,1\right]$$

The opacity head produces a continuous veiling estimate that represents gradual changes during smoke emergence. The first signal summarizes how far the window departs from the scene dynamics prior, as a recency-weighted mean of the calibrated onset score:

(6) $$g_{t,i}^{lev}=\sum_{k=t-W+1}^{t}w_{k}a_{k,i},\;w_{k}\propto \gamma^{t-k},\;\sum_{k}w_{k}=1,\;\gamma \in \left[0,1\right]$$

It captures sustained abnormality while the weights $w_{k}$ let recent evidence dominate, so a level that is still rising is not diluted by the calmer start of the window.

Early smoke’s opacity accretes gradually and, in its initial phase, largely monotonically. Clouds brighten and darken, foliage oscillates, and glare pulses, none of which grows monotonically. From the first differences ${∆}_{k,i}\;=o_{k,i}-o_{k-1,i}$ we combine the trend strength with its consistency. The trend is the ordinary least squares slope of the opacity trajectory, and the consistency is a normalized sign agreement of the increments:

(7) $$\beta_{t,i}=\frac{\sum_{k}\left(k-\bar{k}\right)\left(o_{k,i}-{\bar{o}}_{i}\right)}{\sum_{k}{\left(k-\bar{k}\right)}^{2}}$$

(8) $$m_{t,i}=\frac{1}{W-1}\sum_{k=t-W+2}^{t}sign\left({∆}_{k,i}\right)\in \left[-1,1\right]$$

The growth signal is high only when the opacity is both increasing and doing so consistently:

(9) $$g_{t,i}^{grow}=ReLU\left(\beta_{t,i}\right)\cdot \frac{1}{2}\left(1+m_{t,i}\right)$$

A cloud whose opacity fluctuates yields $m_{t,i}$ ≈0 and is suppressed even when its slope is momentarily positive. Smoke rises and drifts as a spatially coherent field, whereas the two motions that most often masquerade as it, camera shake and swaying foliage, are, respectively, global and oscillatory. Let $v_{k,i}\in R^{2}$ be the per-tile motion estimated from feature space displacement between consecutive frames. We first remove whole-frame ego motion by subtracting the global median flow, which cancels camera shake because it moves every tile alike ${\tilde{v}}_{k,i}=v_{k,i}-{median}_{j}v_{k,j}.$ In the motion-compensated field, we quantify local motion coherence through the magnitude-weighted directional alignment between each tile and its spatial neighbors, ${\bar{v}}_{k,i}=\frac{1}{\left|N\left(i\right)\right|}\sum_{j\in N\left(i\right)}{\tilde{v}}_{k,j}$ so that tiles carrying stronger motion contribute proportionally more to the coherence estimate. Directional persistence is then derived from the ratio of net to total displacement accumulated over the temporal window, $c_{k,i}=cos\left({\tilde{v}}_{k,i},{\bar{v}}_{k,i}\right)\left\|{\tilde{v}}_{k,i}\right\|$ a quantity that approaches unity when motion follows a stable heading and falls toward zero when it meanders and reverses:

(10) $$p_{t,i}=\frac{\left\|\sum_{k}{\tilde{v}}_{k,i}\right\|}{\sum_{k}\left\|{\tilde{v}}_{k,i}\right\|+\varepsilon }\in \left[0,1\right]$$

Oscillatory foliage and camera jitter yield low directional persistence values, since their successive displacements tend to cancel over the temporal window; coherent drift, by contrast, sustains a consistent heading and produces correspondingly higher values. The motion coherence score combines these two complementary signals local directional agreement among neighboring tiles and temporal directional persistence into a single measure, rewarding motion that is coherent both across space and through time:

(11) $$g_{t,i}^{coh}=\left(\frac{1}{W}\sum_{k}ReLU\left(c_{k,i}\right)\cdot p_{t,i}\right)$$

Temporal persistence captures how consistently the calibrated onset score stays above the scene baseline across the observation window. Transient responses, birds crossing the frame, passing vehicles, momentary lens flare, accordingly receive lower persistence scores than the sustained rise that accompanies genuine smoke emergence, which builds gradually and does not subside Table 1. We compute this quantity through a soft occupancy function governed by a temperature parameter, allowing the transition between below and above baseline to be graded rather than thresholder abruptly:

(12) $$g_{t,i}^{pers}=\frac{1}{W}\sum_{k=t-W+1}^{t}\sigma \left(\frac{a_{k,i}-\theta }{T}\right)$$

where σ is the logistic function. A longest run above baseline variant may replace the soft count when flicker rejection must be stricter. Stacking the four signals gives the per-tile emergence descriptor, and the set ${\left\{\phi_{t,i}\right\}}_{i}$ over the tile grid is the emergence field passed to the spatiotemporal aggregation:

(13) $$\phi_{t,i}={\left[g_{t,i}^{lev},g_{t,i}^{grow},g_{t,i}^{coh},\;g_{t,i}^{pers}\right]}^{⊺}$$

The four normalized signals are combined using a conjunctive formulation that requires simultaneous evidence across the emergence cues. The resulting score remains high when all four component values are simultaneously elevated:

(14) $$E_{t,i}=\prod_{s\in \left\{lev,grow,coh,pers\right\}}\sigma \left(\alpha_{s}g_{t,i}^{s}+b_{s}\right)$$

The conjunction is what gives the field its discriminative power. Each distractor is rejected because it fails a different cue, while no single cue is individually decisive, as summarized below.

### 3.3. Temporal Spatial Aggregation

For each frame k in the window ending at t and each tile i, we assemble a token from the instantaneous quantities produced upstream, the backbone feature $z_{k,i}$, the calibrated onset score $a_{k,i}$, the opacity $o_{k,i}$, and the per frame motion alignment $c_{k,i}$, and embed it with learned temporal and spatial position codes:

(15) $$u_{k,i}=W_{in}\left[z_{k,i};\;a_{k,i};o_{k,i};\;c_{k,i}\right]+p_{k}^{t}+p_{i}^{s}\in R^{D}$$

The window level descriptor $\phi_{k,i}$ itself computed causally over the sub-window ending at k, is held back and injected at the head, where it acts as an interpretable prior rather than being diluted through the attention stack. Joint attention overall N × W tokens costs $O\left(N^{2}W^{2}\right)$ and is unnecessary. We factorize into a temporal pass followed by a spatial pass. Both use standard multi-head self-attention, which, for queries, keys, and values Q, K, V and an additive mask M is:

(16) $$Attn\left(Q,K,V\right)=softmax\left(\frac{{QK}^{⊺}}{\sqrt{d}}+M\right)V$$

The temporal attention stage operates independently on the history of each tile under a causal mask, so that the representation at time t draws only on observations available up to t. This constraint preserves the strictly online character of the system: no future frame is ever consulted in forming a present decision, matching the conditions under which early wildfire detection must operate in deployment:

(17) $$M_{{kk}^{\prime }}\left\{\begin{array}{cc} 0, & k^{\prime }\leq k,\\ -\infty , & k^{\prime }>k, \end{array}\right.\;h_{k,i}^{tmp}={\left[Tr{Enc}_{time}\left({\left\{u_{k^{\prime },i}\right\}}_{k^{\prime }=t-W+1}^{t};M\right)\right]}_{k}$$

Here the representation at frame k depends only on frames up to k. The spatial pass then runs per frame over the tile grid, unmasked, letting tiles exchange evidence about plume extent:

(18) $$h_{k,i}^{sp}={\left[Tr{Enc}_{space}\left({\left\{h_{k,j}^{tmp}\right\}}_{j=1}^{N}\right)\right]}_{i}$$

The factorization reduces the cost to $O\left({NW}^{2}d+{WN}^{2}d\right)$ and, because the temporal mask is causal, the keys and values of past frames can be cached so that each new frame is processed in amortized constant time. Caching past keys and values enables causal streaming inference as new frames arrive.

The head input concatenates the aggregated representation with the interpretable descriptor, ${\hat{h}}_{k,i}=\left[h_{k,i}^{sp};\phi_{k,i}\right]$, giving the head direct access to the four emergence signals alongside the learned features. A tile-level head predicts smoke presence per tile, and is supervised wherever tile resolution labels exist:

(19) $$y_{k,i}=\sigma \left(W_{tile}^{⊺}{\hat{h}}_{k,i}+b_{tile}\right)$$

Because an early plume may occupy only a small subset of tiles, image-level prediction uses attention pooling to preserve localized evidence. At ignition, a plume touches only a few tiles, and its signal would be swamped by the many negative tiles. We therefore pool by attention, letting the few informative tiles dominate:

(20) $$\alpha_{k,i}=\frac{exp\left(q^{⊺}{\hat{h}}_{k,i}\right)}{\sum_{j=1}^{N}exp\left(q^{⊺}{\hat{h}}_{k,j}\right)},\;g_{k}=\sum_{i=1}^{N}\alpha_{k,i}{\hat{h}}_{k,i},\;Y_{k}=\sigma \left(MLP\left(g_{k}\right)\right)$$

where q is a learned query. Attention pooling behaves like a soft maximum, so a single strongly emerging region can carry the image-level probability ${\;Y}_{k}$ upward well before the plume is large, the behavior that early detection rewards. The sequence ${\;Y}_{k}$ is the model emergence trajectory; by converting it into an alarm while rejecting isolated spikes, we smooth it, ${\hat{Y}}_{k}=\sum_{j\geq 0}w_{j}Y_{k-j}$ with a short causal kernel ω, and require the smoothed score to hold above a threshold η for ν consecutive frames:

(21) $$t^{*}=min\left\{k:{\hat{Y}}_{k^{\prime }}\geq \eta \;for\;all\;k^{\prime }\in \left[k-v+1,k\right]\right\}$$

The detection delay is then $t^{*}-t_{0}$ relative to the ignition time $t_{0}$, which is the quantity the earliness aware objective minimizes. Here ν is the minimum number of consecutive frames for which the smoothed score must stay above the threshold θ before an alarm is raised. The pair (η, ν) traces the operating curve reported in the evaluation, trading detection delay against false alarms per camera day.

The time to detection (TTD) is a discrete quantity and therefore not differentiable, so training instead uses a soft alarm probability. Let *p_t_* denote the image-level smoke probability at time t and θ the alarm threshold. The relaxed alarm activation is then defined as: $a_{t}=\sigma \left(\frac{p_{t}-\theta }{\tau }\right)$, where *τ* controls how smooth the approximation is. For a positive sequence with ignition at time *t*_0_: $L_{lat}=\sum_{t=t_{0}}^{T}a_{t}\frac{t-t_{0}}{T-t_{0}}$, so that the later an alarm arrives after ignition, the larger the penalty. False alarms on pre-ignition frames and on hard negatives are penalized by: $L_{FA}=-\frac{1}{\left|N\right|}\sum_{t\in N}log\left(1-a_{t}\right)$, where *N* denotes the set of negative frames. The complete objective is:

(22) $$L_{EARL}=L_{det}+\lambda_{lat}L_{lat}+\lambda_{FA}l_{FA}$$

where $L_{det}$ is the tile-level detection loss and the coefficients $\lambda_{lat}$ and $\lambda_{FA}$ balance detection accuracy against the two temporal terms. Raising $\lambda_{lat}$ favors earlier alarms at the cost of more false positives, while raising $\lambda_{FA}$ has the opposite effect, so the pair effectively sets the operating point of the detector.

## 4. Experiments and Results

We evaluate EARL through a set of experiments designed to assess detection accuracy, early warning capability, false alarm behavior, and generalization across different wildfire monitoring environments. FIgLib serves as the basis for supervised training and in-domain evaluation, while PyroNear 2025 and SKLFS WildFire are held out for cross-dataset testing without any supervised retraining, isolating how well the learned representation transfers rather than how well it can be refit. Additional fire-free confusor sequences are used to probe and control false alarms under conditions specifically chosen to be challenging. To guard against scene leakage, we partition the data at the fire event and camera levels rather than the individual frame level, ensuring that no camera or event contributes to both training and testing. The evaluation foregrounds operational metrics such as TTD, detection rate, and false alarms per camera day, alongside conventional detection metrics including precision, recall, F1 score, and mAP. We further conduct ablation experiments to examine the contribution of each main component of the framework.

EARL takes input tiles of 224 × 224 pixels, dividing each frame into a 4 × 4 grid of 16 spatial tiles. A lightweight CNN serves as the feature encoder and produces 256-dimensional representations. Temporal context is modeled over a window of eight frames using a factorized temporal-spatial Transformer. Both the temporal and spatial modules use two layers with four attention heads, a hidden dimension of 256, a feed-forward dimension of 512, and dropout of 0.1. The EMA target encoder uses a momentum coefficient of 0.99. We train with AdamW, using a batch size of 16 sequences, an initial learning rate of 1 × 10^−4^, weight decay of 1 × 10^−4^, and cosine decay over 80 epochs. In the earliness objective, the relaxation temperature is τ = 0.1, and the latency and false alarm terms are weighted by $\lambda_{lat}$ = 0.5 and $\lambda_{FA}$ = 0.3. At inference, the alarm threshold is θ = 0.5, and the score must exceed it on three consecutive frames before an alarm is raised. The full model has roughly 4.9 million trainable parameters.

### 4.1. Datasets and Splits

Our protocol assigns each dataset one of three roles. FIgLib [1] is the primary source for supervised training and in-domain evaluation, and its fire-free footage additionally supplies the unlabeled corpus for the self-supervised prior. PyroNear-2025 [3] and the SKLFS-WildFire test set [2] are entirely from training and used to measure cross-dataset generalization, the setting in which cameras, geography, and capture pipelines all differ from training and in which most published detectors degrade sharply. A curated hard negative set, drawn from documented false positive collections and known confessor sequences, is used for both the false-alarm training term and the false-alarms per camera-day evaluation metric. The guiding principle across all splits is that partitioning is done by fire event and by camera, never by frame, so that no scene leaks between training and evaluation Table 2.

FIgLib comprises nearly 25,000 labeled images (about 24,800) drawn from 315 fire sequences captured by 101 fixed cameras across 30 mountaintop stations of the HPWREN network in Southern California between 2016 and 2021. Each sequence spans roughly 40 min before to 40 min after the first visible plume, sampled at approximately 60 s intervals for about 81 frames per fire, with images at 1536 × 2048 and 2048 × 3072 pixels. Two properties make it the right primary benchmark for a latency-first method. First, every filename encodes the offset in seconds from the first visible plume, giving an exact ignition-referenced timestamp $t_{0}$ for each frame. Second, the cameras are fixed, and their views persist for years, which is the stationarity the scene dynamics prior assumes. We partition by fire event into roughly 50, 25, 25 train, validation, test following the dataset’s established practice, so that no fire and, wherever the station layout permits, no camera appears in more than one split. Each image is divided into overlapping tiles to preserve small plumes, and tile-level binary labels are derived from the smoke annotations for intermediate supervision. FIgLib’s negative frames, which deliberately include strong glare and misleading haze, double as natural hard negatives.

Generalization is assessed without any retraining on the target domains. PyroNear-2025 [3] provides roughly 50,000 images with about 150,000 annotations spanning 640 wildfires across France, Spain, Chile, and the United States, and, unusually, includes video as well as stills, which is what allows the motion-coherence signal to be exercised on this set. Its authors report a cross-dataset F1 near 60%, confirming that it is a deliberately hard, in-the-wild generalization target. The SKLFS-WildFire test set [2] contributes 50,735 images drawn from 3309 real wildfire videos, of which 340 videos (3588 images) are positive. Its scale and video-derived negatives make it well suited to measuring false alarms at operational volume. We transfer the FIgLib-trained model to both sets for zero-shot cross-dataset evaluation. The only adaptation permitted is label-free recalibration of the per-tile residual statistics on target fire-free frames, since this uses no smoke labels and mirrors how the system would be commissioned on a new camera. We report results both with and without this recalibration to make its effect transparent.

The false-alarm term and the false alarm per camera-day metric are evaluated on a curated set of fire-free but smoke-like sequences. Drifting clouds, forming fog and haze, blowing dust, sunrise and sunset glare, headlights, and rain on the lens. These are assembled from FIgLib negatives, from PyroNear’s documented false positive collections, and from additional confusor sequences, and are kept disjoint from all training frames so that false-alarm performance reflects genuine generalization rather than memorized negatives.

### 4.2. Metrics

We separate metrics into two tiers. The primary tier is operational and latency-first. How quickly a fire is detected, how often it is detected at all, and how many false alarms this costs. The secondary tier is the familiar frame- and tile-level classification and localization metrics, reported only for comparability with prior work. Throughout, the unit of analysis for detection is the fire sequence, not the frame, because operational value accrues per fire. Let the test set contain M fire sequences indexed by f, each with an ignition time $t_{0}^{\left(f\right)}$ and, at a chosen operating point (η, ν), a first-alarm time $t^{*\left(f\right)}$. The headline metric is the delay between ignition and first alarm, reported in minutes:

(23) $${TTD}^{\left(f\right)}=t^{*\left(f\right)}-t_{0}^{\left(f\right)}$$

Averaging this over only the fires that were detected is misleading, because a model can appear fast simply by detecting only the easy, already obvious fires and never alarming on the hard ones. We therefore report three quantities together. Over the detected set $D=\left\{f:t^{*\left(f\right)}exist\right\}$ we give the mean and median, and, to remove the selection bias:

(24) $$\bar{TTD}=\frac{1}{\left|D\right|}\sum_{f\in D}{TTD}^{\left(f\right)}$$

a penalized TTD over all fires in which a miss is charged the full horizon H:

(25) $$pTTD=\frac{1}{M}\sum_{f=1}^{M}min\left({TTD}^{\left(f\right)},\;H\right),\;{TTD}^{\left(f\right)}≔H\;if\;f\notin D$$

The pair TTD, pTTD exposes any gap between fast when it fires and fast in aggregate, which a single number would hide. Complementing latency, we report the fraction of fires detected within a horizon H of ignition, at operationally meaningful horizons:

(26) $$DR\left(H\right)=\;\frac{1}{M}\sum_{f=1}^{M}1\left[0\leq {TTD}^{\left(f\right)}\leq H\right]$$

A system is only as useful as DR(H) at the horizon a responder cares about; detection rate is therefore reported with the detection delay. For fire-free sequences, false alarms are defined at the event level using sustained threshold exceedances. Because the decision rule already requires ν consecutive above-threshold frames, we count each maximal run that satisfies the alarm condition as one event. With $N_{fa}$ such events observed over a total of C camera days of fire free footage. $FA=\;\frac{N_{fa}}{C}\;\left[false\;alarms\;per\;camera\;day\right].$ Counting events rather than frames is essential. A continuous twenty-minute activation caused by the same confusor is counted as a single false-alarm event. TTD and false alarms are jointly controlled by the threshold η raising η delays alarms but suppresses false ones. Reporting either metric at a single η is therefore uninformative. We sweep η and report the curve of points and likewise. For a headline scalar that is honest about the trade-off, we fix a false-alarm budget β and read off the best achievable latency and detection rate at that budget:

(27) $$TTD@\beta =pTTB\left(\eta \beta \right),\;DR@\beta =DR\left(H;\eta \beta \right),\;\eta \beta =min\left\{\eta :FA\left(\eta \right)\leq \beta \right\}$$

Comparing methods at a common budget prevents the usual confound in which one model looks earlier only because it is quietly permitted more false alarms.

For comparability with prior work, we report frame-level and tile-level precision, recall, and F1. With true positives, false positives, and false negatives counted over the relevant units:

(28) $$P=\;\frac{TP}{TP+FP},\;R=\frac{TP}{TP+FN},\;F1=\frac{2PR}{P+R}$$

Frame-level recall must be read with care on ignition-referenced data. Frames immediately after $t_{0}$ carry a positive label while the plume is still barely visible, so a miss there reflects fundamental unobservability rather than model error, another reason TTD, which measures when the plume first becomes detectable, is the more faithful summary.

### 4.3. Results and Analysis

We evaluate EARL with a focus on how early it detects wildfire smoke and how few false alarms it raises in doing so. Most detection benchmarks report frame-level accuracy alone, which says little about operational value, so we track both what the model predicts and how quickly it predicts it, using TTD, F1 score, mean average precision (mAP), recall, and false alarms per camera day. The evaluation proceeds in four parts. We begin by comparing EARL against existing wildfire smoke detectors and several mainstream object detection architectures, which establishes its in-domain accuracy together with its computational cost. Because detection scores can always be traded against false alarms, we next fix a common false-alarm operating point and compare early warning behavior on equal terms. We then turn to previously unseen cameras and environmental conditions through cross-dataset experiments, testing how far the approach generalizes beyond its training distribution. A final ablation separates the four components that drive performance and measures each on its own, namely the self-supervised scene dynamics prior, the emergence field, the earliness-aware loss, and the long-term temporal model.

For a consistent comparison, every reproduced method was trained and evaluated on the same FIgLib partition and with the same evaluation procedure. We split the dataset at the fire-event and camera levels into roughly 50% training, 25% validation, and 25% test, so that no event or camera view appears in both training and evaluation. Preprocessing and test sequences were identical across methods. Detection thresholds were tuned for each model on the validation set alone and then held fixed at test time. TTD, F1, mAP, and false alarms per camera-day follow the same definitions for every method. Since detection latency depends heavily on the alarm threshold, we also compared TTD at a common false-alarm operating point, which keeps a method from appearing faster merely because it is permitted more false alarms.

Table 3 benchmarks EARL against established wildfire smoke detection methods on FIgLib across four metrics: TTD, F1 score, false alarms per camera day, and mAP. EARL attains a TTD of 1.5 min, an F1 score of 84.8%, a false-alarm rate of 0.21 FA/cam-day, and an mAP of 88.3%. The clearest gains emerge in comparison with the PyroNear sequential model. Where PyroNear records a TTD of 2.0 min, EARL detects smoke 0.53 min sooner. This reduction in latency does not come at the expense of reliability: the false-alarm rate nearly halves, falling from 0.39 to 0.21 FA/cam-day, while F1 and mAP both improve modestly, rising from 84.1% to 84.8% and from 86.4% to 88.3%, respectively. These results indicate that EARL sharpens detection latency without sacrificing classification and detection accuracy. We caution, however, that cross-method comparisons of this kind warrant interpretive care, as differences in implementation, training protocols, and evaluation settings inevitably shape the reported numbers Figure 2.

Table 4 situates EARL alongside representative object detection architectures across six dimensions: AP_0.5_, AP_s_, precision, recall, model size, and inference speed. EARL reaches an AP_0.5_ of 88.3% and an AP_s_ of 79.2%, outperforming both YOLOv8 (80.2% and 59.6%) and YOLOv10 (78.8% and 61.5%). The gap is most pronounced on small objects, where EARL’s AP_s_ exceed its closest competitor by nearly 18 points, a margin of particular relevance for early smoke detection, where the target is often faint and spatially small. Precision and recall settle at 85.99% and 83.69%, respectively. These gains are achieved with a compact footprint: the model comprises just 4.9 M parameters and runs at 39 FPS under the reported experimental setting. This suggests that EARL strikes a favorable balance, delivering strong detection performance without incurring high computational cost. As with any runtime comparison, we note that FPS figures are sensitive to hardware, input resolution, and implementation choices, and are best interpreted under matched evaluation conditions.

Table 5 reports cross-dataset generalization for all methods trained on FIgLib and tested on PyroNear-2025 and SKLFS-WildFire without supervised retraining on the target domain. On PyroNear-2025, the conventional detectors reach TTDs between 4.67 and 5.80 min; the best of them is YOLO26, at 4.67 min TTD, 66.5% recall, and 0.96 FA/cam-day. EARL cuts the delay to 3.61 min while raising recall to 69.7% and lowering the false-alarm rate to 0.73 FA/cam-day. Label-free recalibration improves these figures further, to 3.20 min TTD, 72.8% recall, and 0.58 FA/cam-day.

SKLFS-WildFire shows the same pattern. The baselines range from 5.18 to 6.40 min TTD, while EARL reaches 4.08 min with 66.8% recall and 0.82 FA/cam-day. Recalibration lowers the TTD to 3.70 min, raises recall to 69.6%, and reduces the false-alarm rate to 0.66 FA/cam-day. Taken together, these results suggest that EARL’s temporal emergence modeling and scene dynamics prior improve robustness under domain shift, and that label-free recalibration adds further gains without supervised retraining on the target data Figure 3 and Figure 4.

Table 6 isolates the contribution of each principal component of EARL through a series of ablations. The complete model attains 1.5 min TTD, 84.8% F1, and 0.21 FA/cam-day; each variant below removes a single element while holding the rest fixed. Removing the self-supervised prior is costly on two fronts: TTD lengthens to 2.1 min and the false-alarm rate more than doubles to 0.49 FA/cam-day, with F1 slipping to 82.6%. Substituting a recurrent aggregator for the emergence field degrades performance further still, to 2.4 min TTD, 81.9% F1, and 0.58 FA/cam-day, the weakest configuration in the table. Replacing the earliness-aware objective with plain cross-entropy tells a more selective story: TTD rises sharply from 1.52 to 2.2 min, yet F1 barely moves (84.8% to 84.0%). The dissociation is informative; it indicates that the earliness-aware term acts primarily on when smoke is detected rather than on conventional classification accuracy, which is precisely its intended role. Shrinking the temporal window to two frames pushes TTD to 2.7 min and lowers F1 to 82.8%, underscoring the value of longer temporal context. The ablations show that the self-supervised prior, the emergence field, the earliness-aware objective, and temporal context each contribute to EARL’s final performance, though their influence is distributed unevenly across metrics, some bearing chiefly on latency, others on classification and false-alarm behavior Figure 5.

## 5. Conclusions

This study presented EARL, a temporal framework for early wildfire smoke detection from fixed-view camera sequences. The approach brings together four elements, namely a self-supervised scene dynamics prior, an interpretable emergence field built on residual magnitude, monotonic opacity growth, motion coherence, and temporal persistence, factorized temporal-spatial aggregation, and an earliness-aware training objective. What distinguishes the framework is that it models the gradual emergence of early smoke while folding detection latency and false alarm control directly into the learning process rather than leaving them to post hoc evaluation.

On the FIgLib benchmark, EARL reached a time to detection of 1.52 min, an F1 score of 84.82%, 0.21 false alarms per camera day, and a mAP of 88.32%. Cross-dataset evaluation showed that the model retained useful performance when transferred from FIgLib to PyroNear 2025 and SKLFS WildFire without supervised retraining. On PyroNear, it obtained a TTD of 3.61 min, 69.74% recall, and 0.73 FA/cam day before recalibration, with corresponding results of 4.08 min, 66.81% recall, and 0.82 FA/cam day on SKLFS WildFire. Label-free residual recalibration yielded further gains on both target datasets.

The ablation experiments indicate that the individual components act on different aspects of performance. Removing the self-supervised prior raised both detection latency and false alarm frequency, replacing the emergence field with a recurrent aggregator lowered F1 and increased false alarms, and substituting conventional cross-entropy for the earliness-aware loss had a markedly larger effect on TTD than on F1. Shortening the temporal window to two frames produced the largest increase in detection latency among the variants examined, underscoring the value of a longer observation window. These results indicate that combining scene-specific normal dynamics, structured temporal cues, and latency-aware optimization can improve the balance among early detection, detection accuracy, and false alarm control, three objectives that are often in tension. The cross-dataset findings further suggest that these components retain useful predictive capability under changes in camera and environmental conditions, supporting continued investigation of latency-oriented temporal modeling for practical wildfire monitoring systems.

EARL localizes smoke within the camera image through its tile-level predictions, but it does not estimate where the fire is on the ground. This is a practical limitation. In operational wildfire monitoring, an alarm is most useful when it also indicates where the suspected fire is, so that crews can verify it and respond quickly. We therefore plan to extend EARL toward geographic localization by combining the detected image region with the known position and orientation of the fixed camera. Given camera calibration and terrain data, the viewing ray through the detected smoke region can be projected onto the terrain surface to obtain an approximate fire location. Where camera views overlap, observations from several cameras could be combined to refine this estimate. The aim is for future deployments of EARL to deliver not only early detection but also location information that responders can act on.

## Figures and Tables

**Figure 1:**
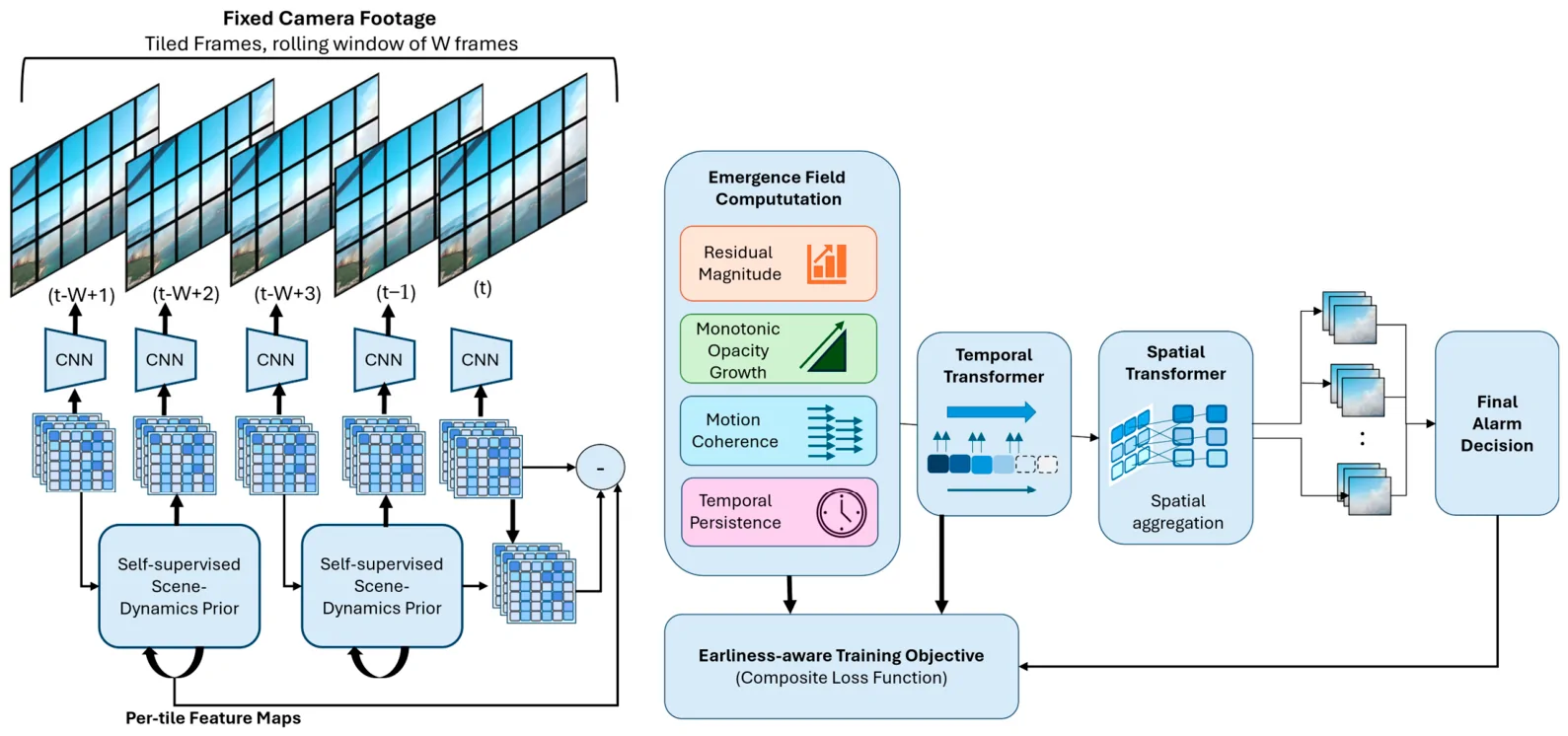
Overall Architecture of the Proposed EARL Framework for Early Wildfire Smoke Detection.

**Figure 2:**
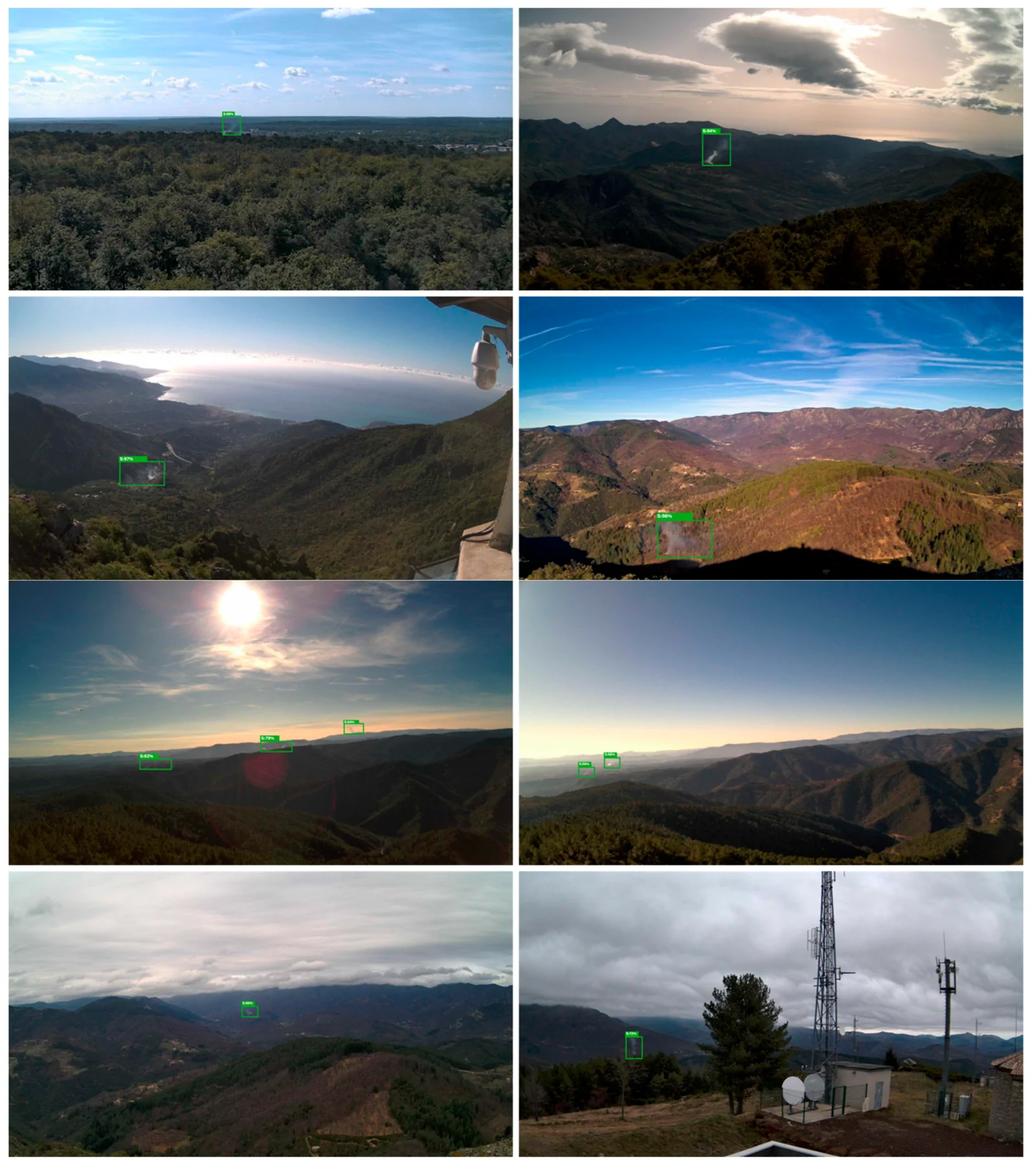
Qualitative examples of early wildfire smoke detection by EARL under diverse environmental and illumination conditions. Green bounding boxes mark the detected smoke regions, with the corresponding confidence score shown above each detection. The examples span clear sky, strong sunlight, cloudy, and complex background scenes, illustrating that the model localizes faint early-stage smoke consistently across settings that differ markedly in lighting and visual clutter.

**Figure 3:**
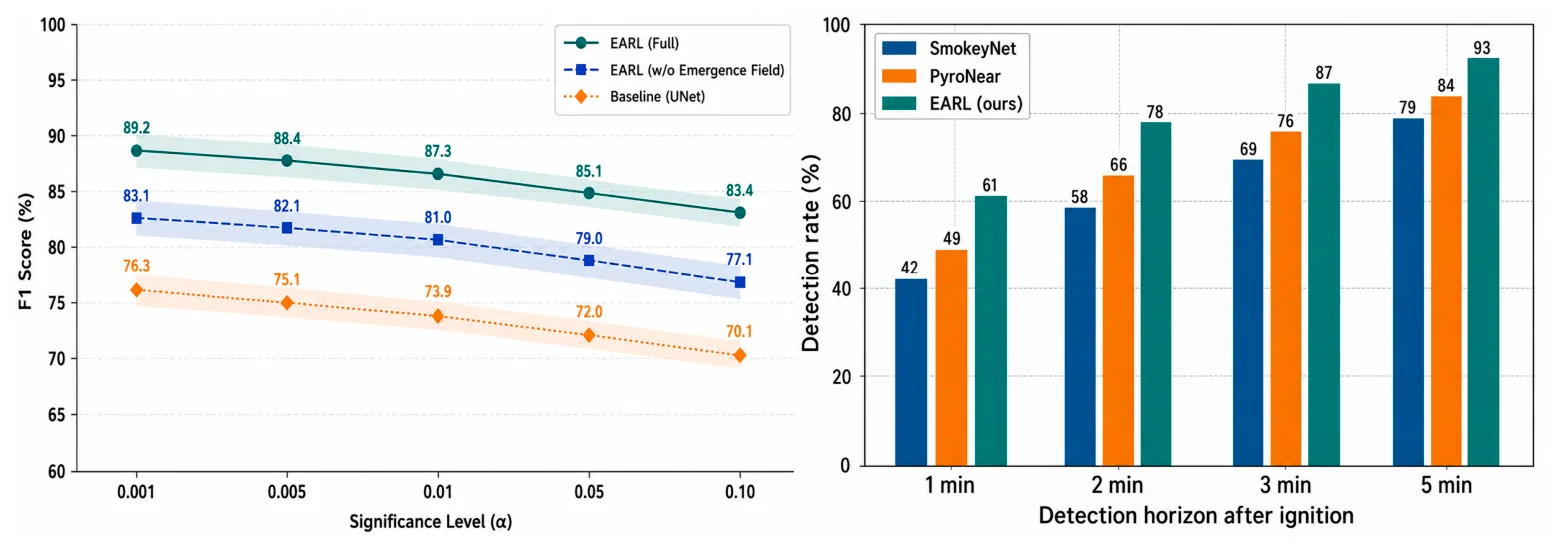

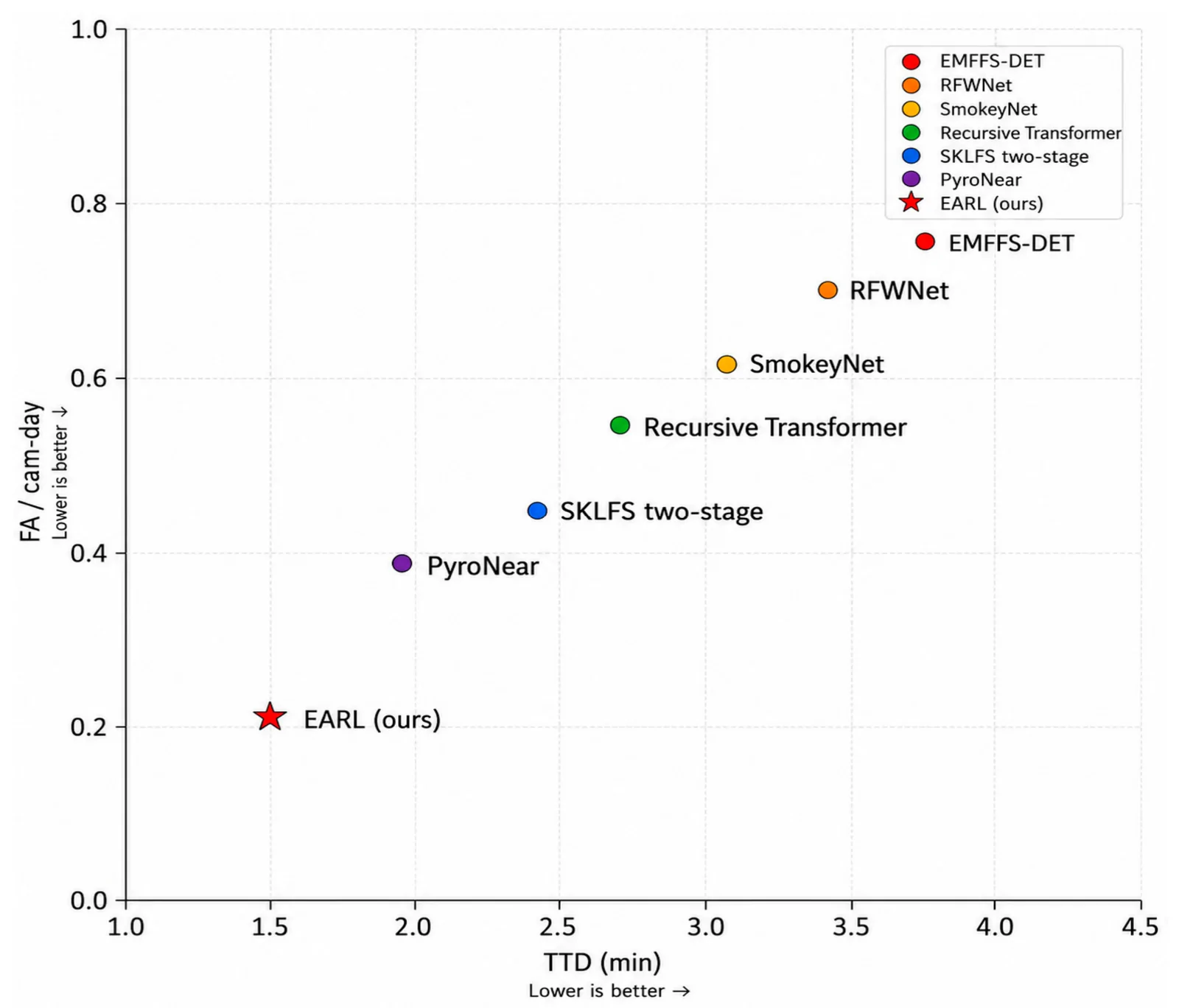
Comparative early detection performance of EARL and baseline methods. TTD versus false alarms per camera-day; detection rate at different post-ignition horizons; comparison of mean TTD and detection rate with statistical analysis.

**Figure 4:**
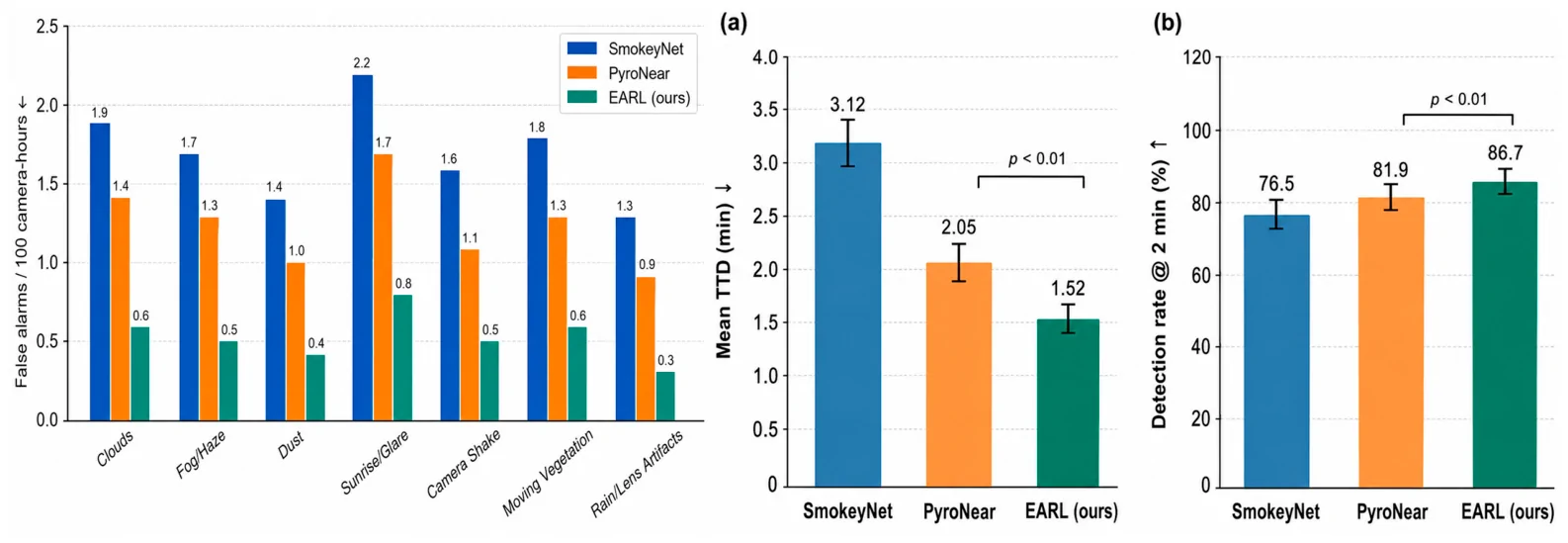
Robustness of EARL under challenging environmental conditions. False-alarm frequency across major confusor categories; TTD, F1, and false-alarm performance under different times of day. (**a**) Mean TTD. EARL achieves the fastest detection, with a mean TTD of 1.52 min, compared with 2.05 min for PyroNear and 3.12 min for SmokeyNet. The reduction in detection time relative to PyroNear is statistically significant (*p* < 0.01), demonstrating that EARL can identify fire/smoke events substantially earlier. (**b**) Detection rate within 2 min. EARL also provides the highest early-detection rate, reaching 86.7%, compared with 81.9% for PyroNear and 76.5% for SmokeyNet. The improvement over PyroNear is statistically significant (*p* < 0.01), indicating more reliable detection during the critical first two minutes following event onset.

**Figure 5:**
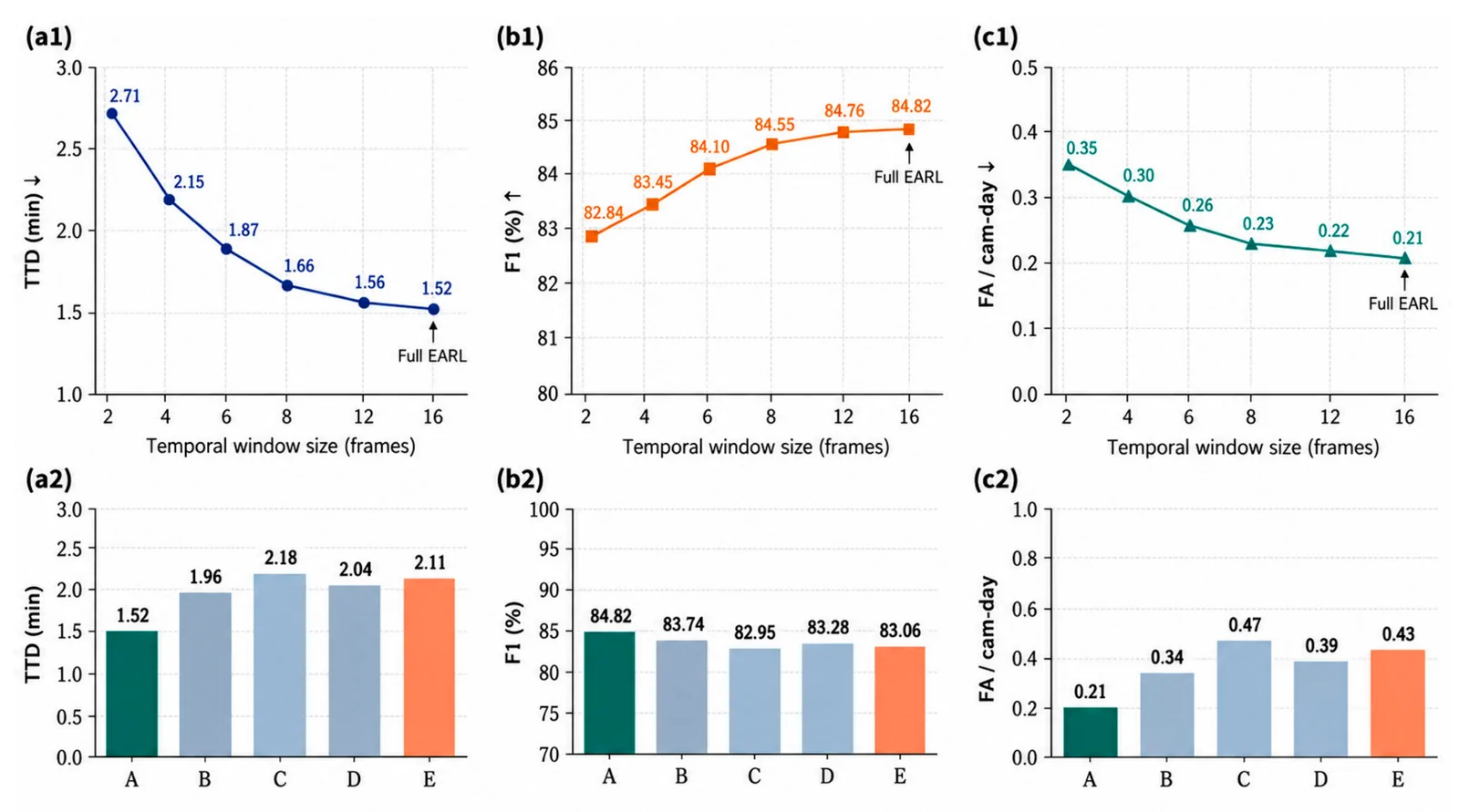
Ablation and sensitivity analysis of the proposed EARL framework. Effect of temporal window size on TTD, F1, and false-alarm rate; contribution of individual emergence field components. (**a1**) mean TTD, (**b1**) F1-score, and (**c1**) false alarms per camera-day (FA/cam-day). Increasing the temporal window progressively improves performance, with the full EARL configuration at 16 frames achieving a TTD of 1.52 min, an F1-score of 84.82%, and a FA/cam-day of 0.21. (**a2**–**c2**) Ablation comparison of configurations A–E in terms of (**a2**) TTD, (**b2**) F1-score, and (**c2**) FA/cam-day. Configuration A, representing the complete EARL model, provides the best overall performance across the three-evaluation metrics. Downward arrows (↓) indicate metrics for which lower values are better, while upward arrows (↑) indicate metrics for which higher values are better.

**Table 1:** Common smoke distractors and the emergence signals they violate.

Distractor	Signal It Fails
Drifting clouds	Monotonic growth
Swaying foliage	Directional persistence and monotonic growth.
Camera shake, pan	Motion coherence and $p_{t,i}.$
Birds, vehicles	Temporal persistence.
Lens flare, raindrops on lens	Temporal persistence and motion coherence.
Sunrise, sunset glare	Suppressed upstream by the time conditioned prior and non-monotonic.

**Table 2:** Summary of datasets and their roles in the experimental evaluation.

Dataset	Role	Scale	Source/Temporal
FIgLib [13]	Train + in-domain test. SSL corpus	≈24,800 images, 315 fires, 101 cameras/30 stations	HPWREN, S. California, ±40 min around ignition at ≈60 s
PyroNear-2025 [22]	Cross-dataset test (has video)	≈50,000 images, ≈150,000 annotations, 640 fires	Web + in-house. FR/ES/CL/US. Images + video
SKLFS-WildFire (test) [21]	Cross-dataset test (large-scale FP)	50,735 images, 3309 videos, 340 positive videos	Real wildfire videos. Video-derived frames
Hard negatives	FA evaluation & training term	Curated fire-free confusor sequences	FIgLib, PyroNear negatives + confusor sequences

**Table 3:** In-domain comparison on FIgLib.

Method	TTD (min) ↓	F1 (%) ↑	FA/Cam-Day ↓	mAP (%) ↑
EMFFS-DET [8]	3.8	78.6	0.78	82.4
RFWNet [9]	3.4	80.1	0.70	84.6
SmokeyNet/FIgLib [13]	3.1	82.5	0.61	80.9
Recursive Transformer [14]	2.7	82.8	0.55	83.7
SKLFS two stage framework [21]	2.3	83.9	0.44	85.9
PyroNear sequential model [22]	2.0	84.1	0.39	86.4
EARL (ours)	1.5	84.8	0.21	88.3

**Table 4:** Performance comparison with mainstream detection models.

Model	AP_0.5_	AP_s_	Precision	Recall	Params/M	FPS
SSD	67.1	37.4	69.2	63.5	26.3	34
Faster R-CNN	71.3	38.5	73.1	64.7	137.1	11
Yolov4	73.4	53.7	73.6	70.6	64.	24
Yolov5	77.6	54.8	75.9	72.3	11.7	29
Yolov7	78.4	56.1	76.8	72.6	3.6	23
Yolov8	80.2	59.6	78.7	73.4	3.0	31
Yolov10	78.8	61.5	77.1	73.2	2.3	31
EARL	88.3	79.2	85.9	83.6	4.9	39

**Table 5:** Cross-dataset generalization without supervised target domain retraining.

Method	Train	Test	Recalibration	TTD (min) ↓	Recall (%) ↑	FA/Cam-Day ↓
SmokeyNet	FIgLib	PyroNear-2025	No	5.80	61.3	1.21
YOLO11			No	5.24	63.8	1.12
YOLOv12			No	4.91	65.4	1.03
YOLO26			No	4.67	66.5	0.96
RT-DETR			No	5.03	64.7	1.07
EARL			No	3.61	69.7	0.73
EARL			Yes	3.20	72.8	0.58
SmokeyNet		SKLFS-WildFire	No	6.40	57.4	1.47
YOLO11			No	5.86	59.5	1.34
YOLOv12			No	5.42	61.7	1.21
YOLO26			No	5.18	62.9	1.14
RT-DETR			No	5.61	60.8	1.27
EARL			No	4.08	66.8	0.82
EARL			Yes	3.70	69.6	0.66

**Table 6:** Ablation study isolating each component’s contribution.

Ablation	TTD (min) ↓	F1 (%) ↑	FA/Cam-Day ↓
Full EARL	1.5	84.8	0.21
− self-supervised prior	2.1	82.6	0.49
− emergence field (plain recurrent)	2.4	81.9	0.58
− earliness loss (cross-entropy)	2.2	84.0	0.27
window = 2 frames	2.7	82.8	0.35

## Data Availability

The datasets used in this study are publicly available from their respective repositories. The FIgLib dataset is available through the HPWREN Fire Ignition Library at https://www.hpwren.ucsd.edu/FIgLib/ (accessed on 10 February 2026). The PyroNear-2025 dataset, including image and video sequences, annotations, and evaluation resources, is available at https://github.com/joseg20/wildfires2025 (accessed on 3 January 2026). https://drive.google.com/drive/folders/1-6NR3M9qBi7FTpEt0rhRQeEWD_33mBGH (accessed on 8 February 2026). The SKLFS-WildFire test dataset is available through the authors’ repository at https://github.com/WCUSTC/CCPE (accessed on 20 January 2026). The curated hard-negative set used in this study was assembled from fire-free and confessor sequences as described in the Experimental Design section.
